# Supplementary figures and images for: IL-17 cytokines preferentially act on naïve CD4+ T cells with the IL-17AF heterodimer inducing the greatest functional changes
Source: PLoS One. 2023 Apr 28;18(4):e0285166. doi: 10.1371/journal.pone.0285166 (PMC10146571; doi:10.1371/journal.pone.0285166)

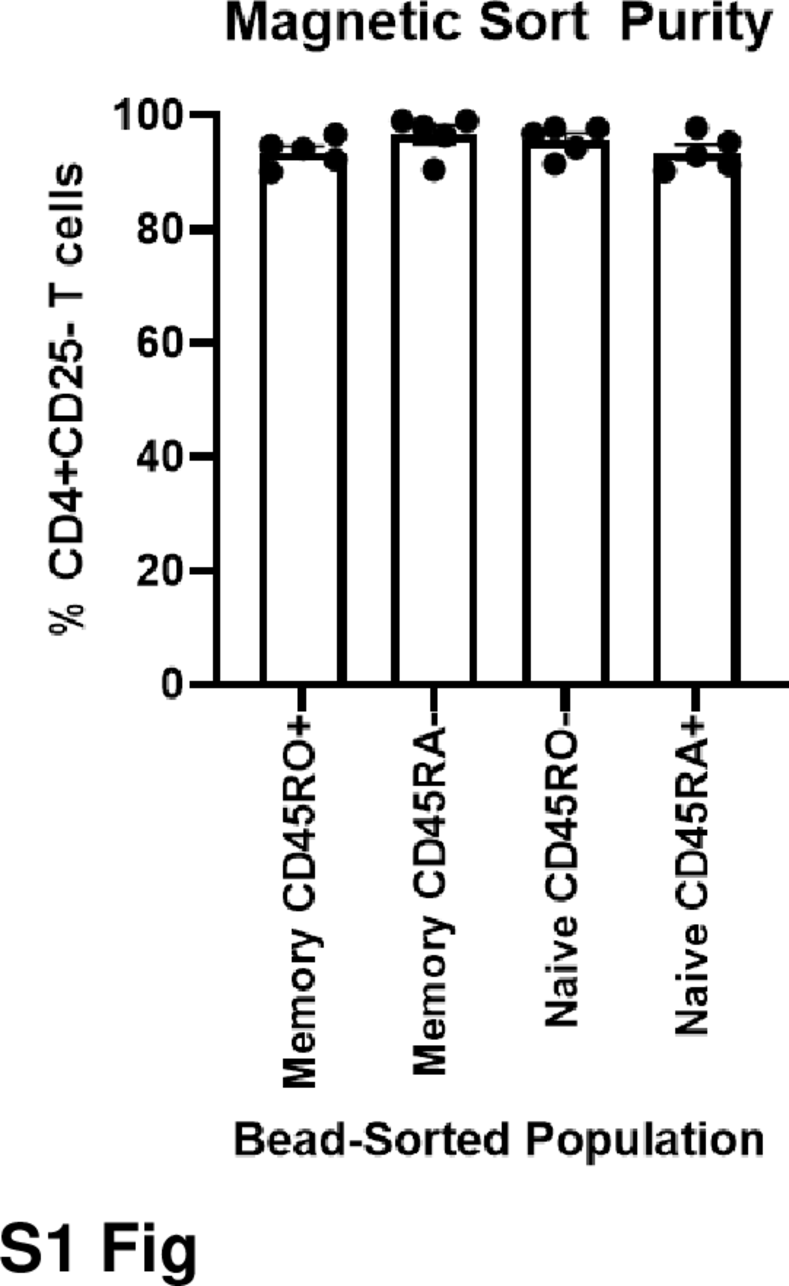

Supplement: S1 Fig — Ex vivo CD4+25- bulk cells were magnetically sorted with either CD45RO+ or CD45RA+ Miltenyi positive selection beads. Mean purity of the sorts is 93.62% +/- 1.13; N = 5. (TIFF) [file pone.0285166.s001.tiff]

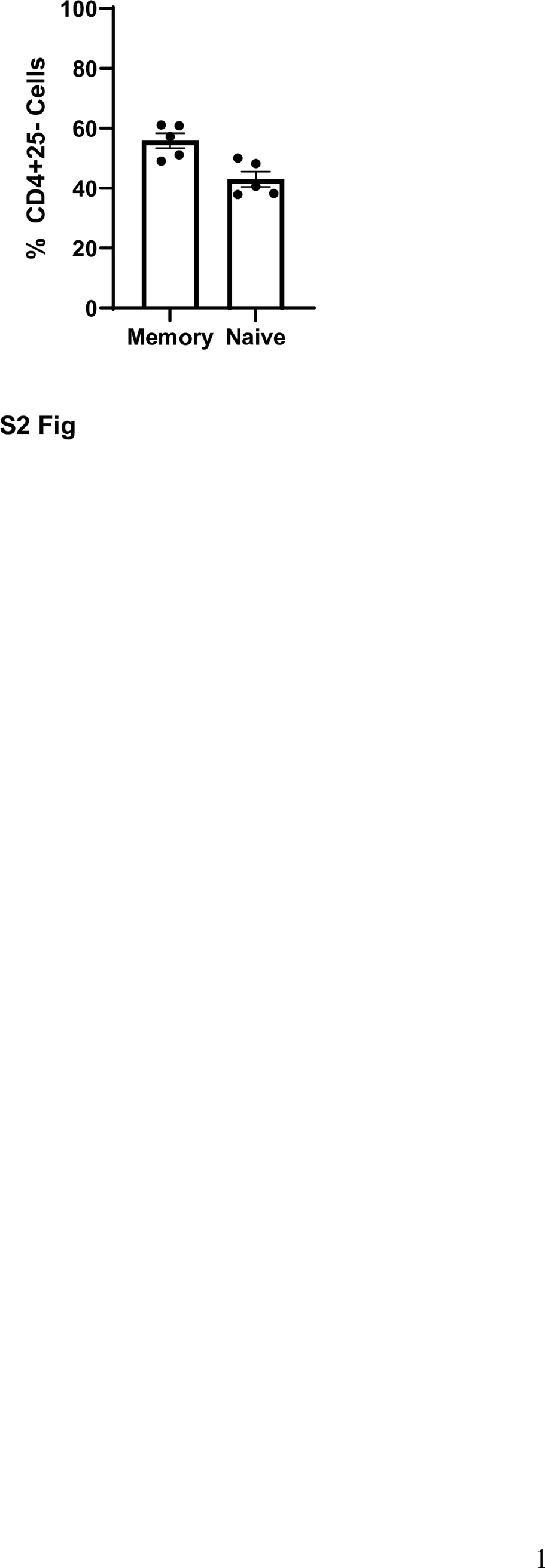

Supplement: S2 Fig — The bulk ex vivo CD4+25- T cell populations were made up of ~57–58% memory and ~42–43% naïve T-cells; N = 5. (TIFF) [file pone.0285166.s002.tiff]
